# The role of bromeliad structural complexity on the presence, spatial distribution and predator avoidance in *Tityus neglectus* (Scorpiones: Buthidae)

**DOI:** 10.1002/ece3.11522

**Published:** 2024-06-04

**Authors:** Maria Carolina de Oliveira Souza, Stênio Ítalo Araújo Foerster, Renato Portela Salomão, João Pedro Souza‐Alves, Geraldo Jorge Barbosa de Moura, André Felipe de Araujo Lira, Rodrigo Barbosa Ferreira

**Affiliations:** ^1^ Departamento de Biologia Universidade Federal Rural de Pernambuco Recife Brazil; ^2^ Departament of Zoology University of Tartu Tartu Estonia; ^3^ Facultad de Estudios Superiores Iztacala Universidad Nacional Autónoma de México Tlalnepantla de Baz Mexico; ^4^ Centro de Biociências, Departamento de Zoologia Universidade Federal de Pernambuco Recife Brazil; ^5^ Colección Nacional de Arácnidos Instituto de Biologia, Universidad Nacional Autónoma de México Ciudad de México Mexico; ^6^ Programa de Pós‐Graduação Em Biologia Animal, Departamento de Ciências Biológicas Universidade Federal Do Espírito Santo Vitória Espírito Santo Brazil

**Keywords:** defensive behaviour, neotropical arachnids, plant–arthropod interaction, population ecology

## Abstract

The spatial arrangement of organisms is significantly influenced by the structure of vegetation. Bromeliads, characterized by a remarkable architectural design featuring rosette‐like leaf arrangements for rainwater storage, act as habitats for various organisms. These organisms use bromeliads for shelter, foraging, reproduction and the supply of nutrients and moisture. This study investigated how specific aspects of bromeliad structure, such as the number, width and length of leaves, impact the behaviour and distribution patterns of the bromelicolous scorpion *Tityus neglectus*. In the examination of 110 sampled bromeliads, 33 scorpions were recorded, resulting in an occupancy rate of 30%. The likelihood of scorpion occurrence was associated with the plant's structure. The length and coefficient of variation in the width of leaves appeared as the main predictors, positively influencing scorpion presence while the number of leaves exhibited a negative relation with scorpion occurrence. The distribution of scorpions was uniform across the spatial design of bromeliads. Furthermore, *T. neglectus* demonstrated the ability to utilize water accumulated in the bromeliad to evade potential predators, submerging itself for, on mean, almost 8 min. We concluded that bromeliad structure is essential in shaping the distribution patterns and anti‐predatory behaviour of *T. neglectus*.

## Graphical abstract

Due to remarkable architectural design featuring rosette‐like leaf arrangements, bromeliads serve as habitats for various species. Bromeliad architecture is a key factor in the spatial distribution and occurrence of the *Tityus neglectus* scorpion. Furthermore, *T. neglectus* is able to use the water accumulated in the phytotelm to escape potential predators.

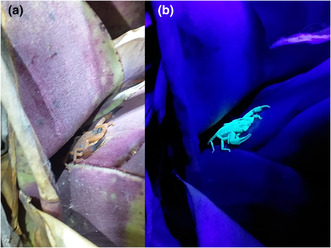

## 1 INTRODUCTION

The spatial distribution of an organism is crucial for its successful establishment in the ecosystem. Individual fitness and survival are expected to increase with selection of sites with low predation risk and reduced competition (Huey, 1991; Vollrath, 1987). Structurally complex habitats may increase food availability, provide shelter against predators and refuge from climatic harshness and supply alternative resources (Norbury & Overmeire, 2018; Warfe & Barmuta, 2004). For example, phytotelm‐associated arthropods benefit from increasing plant architectural complexity (Ferreira et al., 2020; Redi & Hochuli, 2007; Vasconcelos‐Neto et al., 2017). Therefore, a fine scale exerts a key role for phytotelm‐associated arthropods that depend on the architecture of their host plants (Gonçalves‐Souza et al., 2015).

The unique architecture of plants from the Bromeliaceae is essential for many arthropods (Dias et al., 2014; Pereira & Quirino, 2008). Due to the complexity of their structure, the bromeliads offer all those features found in this type of habitat (see Dias & Brescovit, 2004; Hernández‐Baz et al., 2011; Panizon et al., 2014). Previous studies indicated that bromeliads are biodiversity amplifiers and key elements in the structuring of communities (Gonçalves‐Souza, Brescovit, et al., 2010; Jorge et al., 2021; Laviski et al., 2021). The spiral arrangement of their leaves forms a rosette‐like structure that can store water and organic debris in the form of phytotelm (Cogliatti‐Carvalho et al., 2010; Islair et al., 2015; Kitching, 2001; Rocha, 2002). These bromeliad traits create both an aquatic and terrestrial ecosystem on a small spatial scale (Ladino et al., 2019).

Arthropods associated with bromeliads may use the phytotelm water to escape from predators, as reported for spiders of the genus *Corinna* Kocj, 1841, and *Coryphasia* Simon, 1902 (Piccoli, 2011; Romero et al., 2007). The reduced habitat availability in these bromeliads may increase the interspecific competition, thus it is crucial to determine the distribution of species in this microenvironment. Previous studies have suggested that larger bromeliads can host high abundance and diversity of arthropods, due to the increased number of microhabitats (Araújo et al., 2007; Peterman et al., 2015). This high abundance of invertebrates may also attract predators to bromeliads, which influence bromeliad community (Breviglieri & Romero, 2017; Hammill et al., 2015; Peterman et al., 2015). In fact, the absence of larger‐bodied predators in bromeliads increases the abundance of small‐bodied mesopredators (Breviglieri & Romero, 2017). Therefore, predator avoidance may appear determinant for the distribution of invertebrates within bromeliads.

Scorpions from the families Vaejovidae, Bothriuridae, Chactidae and Buthidae have been recorded using bromeliads (Francke & Boos, 1986; Mondragón & Ruiz, 2009; Ochoa et al., 1993; Santos et al., 2009). *Vaejovis franckei* Sissom, 1989, may use *Tillandsia* sp. as shelter in a subtropical rainforest in Oaxaca, southern Mexico (Mondragón & Ruiz, 2009). *Tityus neglectus* Mello‐Leitão, 1932, was often sheltering in terrestrial bromeliads (*Aechmea* spp. and *Hohenbergia* spp.) in northeastern Brazil (Santos et al., 2003, 2006). Additionally, it is suggested that *T. neglectus* is able to use the phytotelm of these terrestrial bromeliads as an escape route (Lira, A.F.A., pers. obs.). In general, scorpions are territorial predators with limited dispersal ability that can be found in microhabitats near their potential prey (Polis, 1990; Polis et al., 1985). In addition to their role as predators, scorpions are usually listed as preys of larger predators such as vertebrates and tarantulas (Polis et al., 1981). Therefore, bromeliads may also be an important shelter for scorpions due to the moisture and availability of potential prey as well as provide refuge from larger predators.

In this context, we aimed to analyse the role of bromeliad structure on *T. neglectus* scorpions in a humid forest enclave in Northeastern Brazil. We predicted that bromeliads with more complex architecture (i.e. high number of larger and longer leaves) would favour the presence of scorpions. Large bromeliads increase the number of microenvironments (spatial niche) reducing interspecific competition (Srivastava, 2006). Also, we predicted that bromeliads aggregated distribution would enhance the presence of scorpions. Because of the low dispersal capability of scorpions, the location of the bromeliads close to each other may facilitate the colonization of neighbouring bromeliads. Finally, considering our observation of potential use of bromeliads as a refuge for scorpions, we tested the scorpions' ability to stay submerged in phytotelm to escape from predators.

## 2 MATERIALS AND METHODS

### 2.1 Study area

**FIGURE 1:**
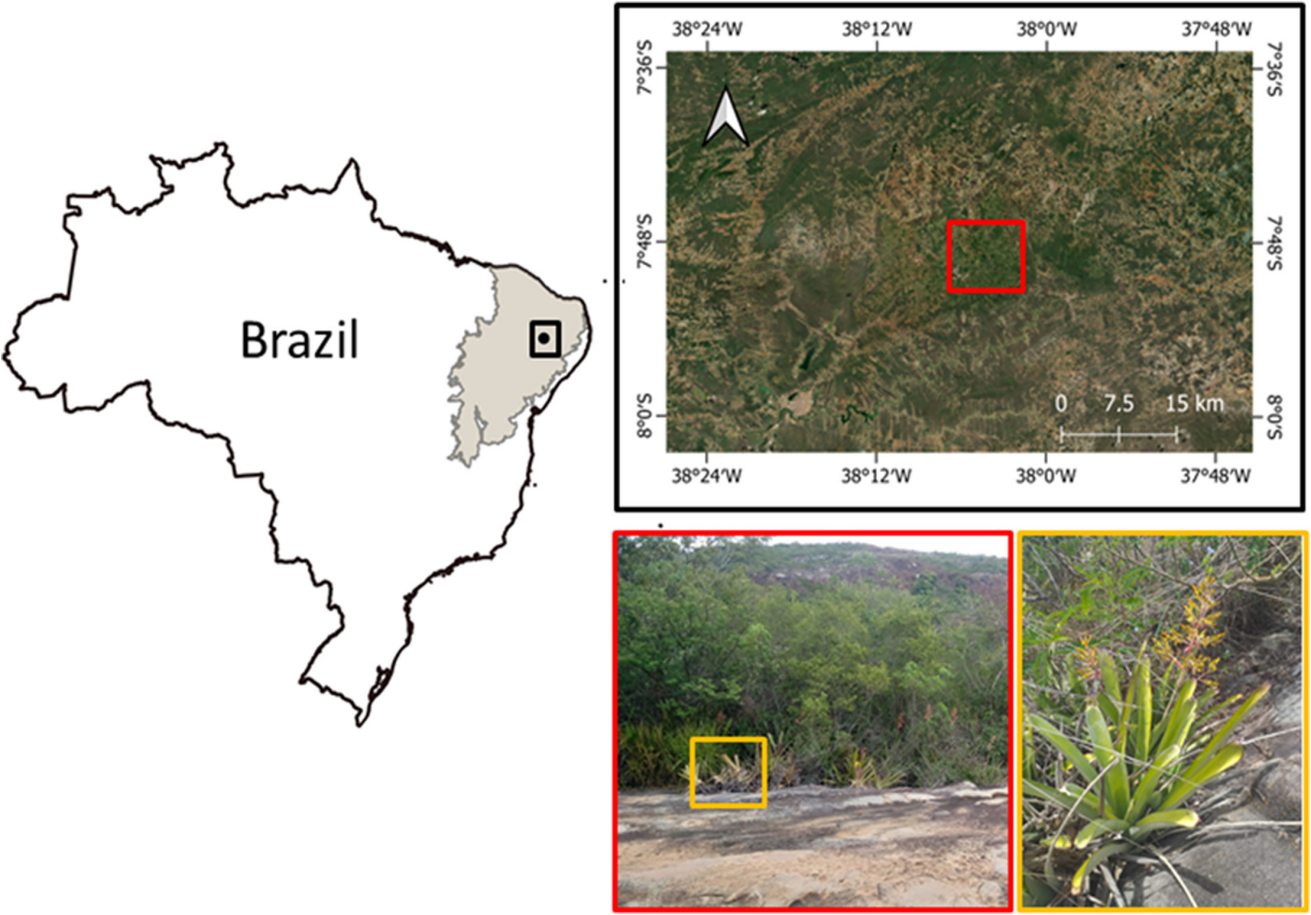
Location of study area located in Triunfo municipality, Pernambuco, Brazil. Sampled rocky outcrop (red square) with *Aechmea leptantha* patches (orange square).

The fieldwork was conducted in January 2020 at two rocky outcrops dominated by the tank bromeliads *Aechmea leptantha* (Harms) Leme & J.A. Siqueira in Northeastern Brazil (38°05′ W, 07°50′ S) (Figure 1). The study area is classified as a humid forest enclave known as ‘Brejo de Altitude’ (Andrade‐Lima, 1966). The area is inserted in the matrix of undisturbed and secondary forests, rocky outcrops, monocultures (e.g. coffee) and livestock activity. The annual rainfall ranges from 634 to 1190 mm^3^; the mean annual temperature is 25 ± 13.7°C (Karger et al., 2017). The region has a dry season from August to October, and a rainy season from February to April (Climate‐Data, 2023).

### 2.2 Field data collection

**FIGURE 2:**
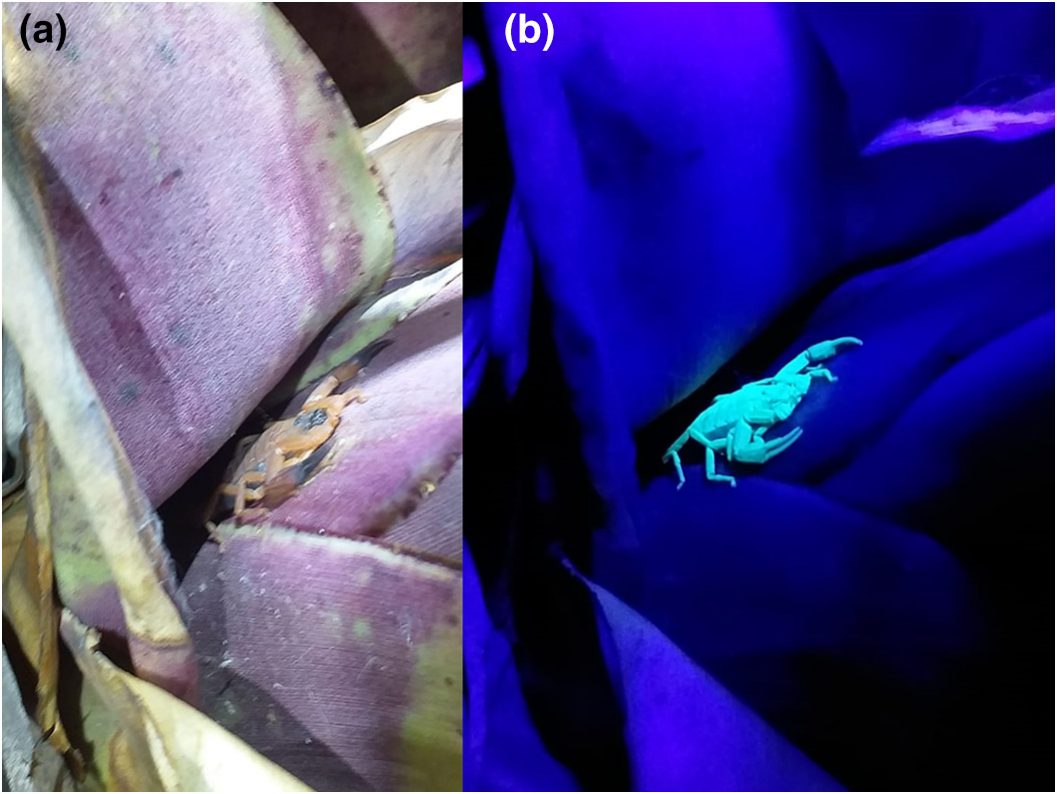
*Tityus neglectus* Mello‐Leitão, 1932, scorpion found inside *Aechmea leptantha* bromeliad on normal (a) and ultraviolet light (b).

In order to characterize the bromeliad structure, we measured the following variables: number and measure (width and length) of leaves. Firstly, we counted the number of leaves in each bromeliad. Secondly, we measured the width and length of bromeliad leaves in millimetres. The width of the leaves was measured in their median portion because it was the maximum height where the scorpions were found. Such measures were performed using a calibrated tape in millimetres. A total of 110 bromeliads (*n* = 55 individuals/outcrop) were randomly chosen from the two rocky outcrops. To verify the presence/absence of scorpions in these bromeliads, we conducted nocturnal survey (19:00–22:00 h). As scorpions are sensitive to ultraviolet light (see López‐Cabrera et al., 2020), we used ultraviolet flashlights to facilitate visualization of the individuals on the bromeliads (Figure 2). Finally, we georeferenced the location of each sampled bromeliad. The identification of the individuals was based on Lourenço and von Eickstedt (1988).

### 2.3 Submersion behaviour trials

The scorpions were taken to a provisory laboratory established near the collection area. To simulate a bromeliad, we created an artificial microecosystem using a transparent plastic terrarium containing 300 mL of bromeliad water (ca. 10 cm of water column). In these microsites, we placed a leave of *Aechmea leptantha* (±15 cm in length) displaced at an approximate angle of 45° inside the terrarium. With the help of metal tweezers, the scorpions were gently positioned on the out‐of‐water part of the adaxial surface of the leaf and left to acclimatize for 5 min. After this period, to stimulate the diving behaviour of the scorpions in response to a potential threat, we slightly touched the mesosoma of the individual using a metal tweezer (30 cm). If the individuals submerged after the touch, we counted the time (in seconds) until the complete return of the individual out of the water column. We considered a complete submersion when the individuals inserted the whole body under the water. We also tested submersion behaviour with a non‐bromeliad, congeneric scorpion, *Tityus pusillus* Pocock, 1893, to verify if diving behaviour is related to bromelicolous habit.

Because scorpions are nocturnal predators (Polis, 1990), we experienced each individual once during the night (23:00–01:00) in a darkroom with the aid of red light (Machan, 1968). After each round of trial, we replaced the water, leaves and the terrarium. Voucher specimens were deposited in the Arachnological Collection of Universidade Federal de Pernambuco, Brazil.

### 2.4 Data analysis

The effect of structural elements of bromeliad plants on the occurrence of *T. neglectus* was initially examined using mixed logistic models fitted with the lme4 R package (Bates et al., 2015). We included the sampling sites as a random effect to account for potential pseudo‐replication issues due to spatial autocorrelation in the occurrence of *T. neglectus*. However, the standard deviation of the site group was 0 in all cases (i.e. full and reduced model, see below), indicating that no variation could be attributed to the random effect. In this scenario, the parameter estimates converge to what would be obtained in a binomial generalized linear model (i.e. no random effect). Thus, the results presented here are based on binomial GLMs.

An initial binomial GLM was fitted using the presence/occurrence of *T. neglectus* in bromeliad plants as the response variable, and all bromeliad traits as predictors (full model). The bromeliad traits (predictors) included the number of leaves, average leave length and width (cm) and the coefficient of variation of leave length and width (cm). The full model was subsequently optimized into a reduced model using the automatic model reduction based on AIC scores implemented in the stepAIC function (argument direction = ‘both’) of the MASS R package (Venables & Ripley, 2002). Multicollinearity in predictor variables was quantified using variance inflation factor (VIF) scores calculated with the car R package (Fox & Weisberg, 2019). Odds ratios and their respective 95% confidence intervals were calculated for each predictor using the parameters R package (Lüdecke et al., 2020) and interpreted as effect sizes in the context of our analysis.

To determine the spatial distribution of *T. neglectus* over the bromeliads, we first plotted the geographic location of each animal on a 30 cm × 30 cm grid using QGis v. 2.14.10 software (QGis, 2023). We chose this grid size due to low ability of dispersal of the scorpions. We then counted the number of scorpions in each cell and calculated the variance for the mean ratio index (Neumann & Starlinger, 2001). A value of 1 indicates a random distribution, values <1 indicate a uniform distribution and values greater than 1 indicate a clustered distribution (Neumann & Starlinger, 2001).

To compare the diving behaviour of *T. neglectus* individuals with that of *T. pusillus*, a generalized linear model with negative binomial distribution was used, given that the model showed a high overdispersion (Residual deviance/Residual d.f. >2). Individuals were used as predictor variables, and latency to start moving underwater (recorded in seconds) was used as response variable. We tested the residual normality using normal Q–Q plots and the presence of outliers was evaluated, but none was found (Cook's distance >1). This analysis was performed with the MASS package (Ripley et al., 2018) in software R version 3.2.0 (R Core Team, 2023).

## 3 RESULTS

**FIGURE 3:**
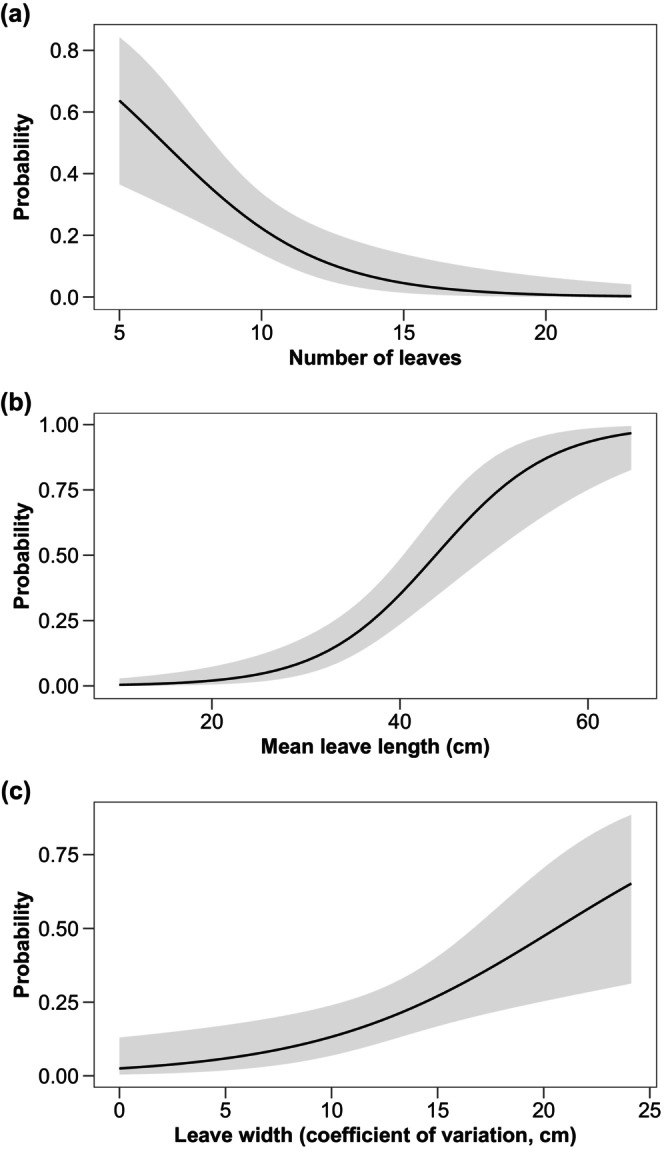
Predicted probability of finding the scorpion *Tityus neglectus* Mello‐Leitão, 1932, as a function of structural elements of *Aechmea leptantha* bromeliad in Northeastern Brazil. Predictions are based on a multivariate logistic regression model using the presence/absence of scorpions as the response variable. Only the statistically significant predictors from the reduced model are shown; see Table 1 for details.

We found 33 individuals of *T. neglectus* (males = 12, females = 12, juveniles = 9) from 110 sampled bromeliads, resulting in 30% of frequency of occurrence. The bromeliads varied in structure, with a mean number of 11 ± 4 leaves (range: 5–23) with mean length and width of 34.68 ± 10.61 cm (range: 10.16–64.69 cm) and 5.19 ± 0.80 cm (range: 2.75–7.40 cm), respectively. The presence of *T. neglectus* was influenced by bromeliad architecture (Table 1), with a negative probability of occurring in bromeliads with more leaves (Figure 3a). However, the occurrence of *T. neglectus* was responsive to the length of leaves. Bromeliads with larger leaves had higher probability of *T. neglectus* occurrence (Figure 3b). Lastly, coefficient of variation of leaf width was positively related to the occurrence of scorpions in bromeliads (Figure 3c). Only one scorpion was found per plant. According to the distribution pattern index, the bromeliads with scorpions had uniform distribution (*R* = 0).

**TABLE 1:** Effect of structural elements of bromeliad plants in the occurrence of the scorpion *Tityus neglectus* in Northeastern Brazil estimated by logistic regression.

Model		Est.	2.5%	97.5%	*p*	OR [95% C.I.]	VIF	AIC
M1	Intercept	−6.38	−11.21	−2.19	<.01	0.22 [0.11, 0.39]		94.4
	Number of leaves	−0.36	−0.58	−0.17	<.01	0.27 [0.12, 0.54]	1.51	
	Mean leave length (cm)	0.16	0.10	0.23	<.01	5.54 [2.84, 12.05]	1.88	
	CV leave length (cm)	−0.02	−0.06	0.03	.28	0.77 [0.48, 1.46]	1.08	
	Mean leave width (cm)	0.31	−0.43	1.09	.42	1.28 [0.71, 2.42]	1.06	
	CV leave width (cm)	0.17	0.05	0.31	<.01	2.24 [1.25, 4.27]	1.33	
M2	Intercept	−5.44	−8.64	−2.70	<.01	0.23 [0.12, 0.40]		92.2
	Number of leaves	−0.36	−0.59	−0.17	<.01	0.27 [0.12, 0.53]	1.54	
	Mean leave length (cm)	0.16	0.10	0.24	<.01	5.68 [2.95, 12.3]	1.77	
	CV leave width (cm)	0.18	0.06	0.31	<.01	2.32 [1.34, 4.32]	1.25	

*Note*: The presence/absence of *T. neglectus* was entered as the response variable in a full model (M1) containing all predictors (see ‘Section 2’), which was automatically optimized (M2) to retain the predictors yielding the best fit to the data (i.e. lower AIC score). Odds ratios (OR) with their respective 95% confidence intervals and the variance inflation factor (VIF) scores were calculated for each predictor.

Abbreviations: Est., estimate; CV, coefficient of variation; SE, standard error.

Regarding diving behavioural experiment, the time spent underwater varied between *Tityus* species (*F*_1,37_ = 43.47; *p* < .01). Upon entering the water, individuals of *T. neglectus* formed a curvature of the mesosoma, which stored an air film in their ventral region. While submerged, individuals of *T. neglectus* moved either to the abaxial surface of the leaf or to the bottom of the terrarium and remained motionless for a mean of 7.75 ± 7.68 min (ranging from 1 to 25 min). In contrast, individuals of *T. pusillus*, upon entering the water, continued to flee, foraging on the leaf and remaining submerged for a mean of 18.20 ± 15.56 s (ranging from 3 to 72 s).

## 4 DISCUSSION

We studied the effects of bromeliad architecture on the occurrence, distribution pattern and threat avoidance of the scorpion *T. neglectus*. Our findings indicated that these scorpions have an intrinsic relationship with bromeliads. Bromeliad architecture was a determinant of the presence of *T. neglectus*. The occurrence of scorpions was negatively related to the number of leaves. In contrast, the coefficient of variation of width and length of leaves showed a positive relation with the presence of scorpions. Furthermore, we observed a uniform distribution of the bromeliads harbouring scorpions. Also, *T. neglectus* submerged underwater to avoid eventual threats. Complex microenvironments such as bromeliads are prone to increase the diversity of spatial niches (Srivastava, 2006), which leads to reduced intra‐ and interspecific competition for space and food resources (Gonçalves‐Souza et al. 2010a, 2010b).

Our results indicated a negative effect of the number of leaves on the occurrence of *T. neglectus*. An increase in habitat complexity reduces the encounter with potential prey (Günther et al., 2014; Norbury & Overmeire, 2018; Srivastava, 2006). Considering that scorpions are predators that use the ‘sit‐and‐wait’ hunting strategy (Polis, 1990), the increased complexity of bromeliads could decrease the chances of *T. neglectus* finding potential prey, as described in other bromeliad predators such as damselfly larvae (Srivastava, 2006). Another non‐exclusive explanation may be that bromeliads with most leaves may attract and support larger predators (Gonçalves‐Souza et al. 2010a, 2010b; Peterman et al., 2015) that may compete and prey on scorpions. For example, tarantulas are large invertebrates that are commonly found in bromeliads (Frank & Lounibos, 2009), and they are efficient predators of scorpions (Dor et al., 2011; Duberstein & Sherwood, 2020).

We also found a higher likelihood of *T. neglectus* occurrence in plants with longer leaves. The *A. leptantha* bromeliad possesses serrated leaves (Maciel et al., 2015) that may be related to scorpion protection against predators allowing them to hide deeper into the plant. Longer leaves were also the predictor of jumping spider *Psecas chapoda* (Peckham & Peckham, 1894) occurrence in *Bromelia balansae* Mez (Romero & Vasconcellos‐Neto, 2005). It is because the funnel shape of *B. balansae* associated with thorns at the edges of its leaves becomes an ideal refuge for protecting spiders from potential predators. Previous studies pointed out those rosette‐shaped plants, especially with thorns, are an important refuge for many invertebrates by preventing predation (Bomfim et al., 2021; Cobbold & O'Donnell, 2021; Schmitz et al., 2023). In addition, we found that the coefficient of variation of width in the leaves is positively related to *T. neglectus* presence in the bromeliads. Bromeliads have higher variability regarding morphological traits according to environmental conditions (e.g. Barberis et al., 2017; Cach‐Pérez et al., 2018; Rapnouil et al., 2023). For example, *A. aquilega* (Salisb.) Griseb. exposed to high light intensity possess smaller, wider and more vertical leaves than individuals exposed to lower light intensity (Rapnouil et al., 2023). Therefore, the phenotypic plasticity of bromeliads may add a factor to environmental complexity. It is plausible to argue that the variation in the width of the leaves of *A. leptantha* may benefit *T. neglectus* by increasing the complexity of the environment and preventing large predators.

*Tityus neglectus* exhibited uniform distribution throughout the studied sites. The dynamics of environmental occupation result from processes that directly influence population demography (Hortal et al., 2010). Therefore, a uniform distribution pattern is typically a consequence of direct competition, either intra‐ or interspecifically (Grohmann et al., 2010; Mueller‐Dombois & Ellenberg, 1974; Taylor, 1984). Scorpions are generalist predators that typically exhibit aggressive behaviour towards conspecifics and other species, resulting in intraguild and cannibalistic predation (Dionisio‐da‐Silva et al., 2024; Polis & McCormick, 1987). Thus, the uniform distribution pattern found in *T. neglectus* may be a result of competition for shelter in bromeliads. The single individual found per bromeliad reinforces this species is highly territorial. Nonetheless, it is important to consider that bromeliads have patchy spatial distributions, which may influence their host distribution (Cascante‐Marín et al., 2006; Céréghino et al., 2020; Romero & Vasconcellos‐Neto, 2005). In the case of *A. leptantha*, the distribution in clutches comprised different spatial configurations, with some bromeliad clutches more grouped than others, as well as bromeliad clutches that were closer to forested patches, while others were in the middle of rocky outcrops. Spatial mechanisms that drive *T. neglectus* to exhibit uniform distribution throughout bromeliads need to be more carefully analysed, aiming to disentangle the potential effects of habitat structure and competition among individuals that inhabit these plants.

To our knowledge, here we presented the first record of diving behaviour for Neotropical scorpions. When threatened *T. neglectus* dove into the water stored in the bromeliads. The ability to submerge in phytotelm in response to a threat is known for bromeliad spiders (Hénaut et al., 2018; Piccoli, 2011; Romero et al., 2007). For example, *Cupiennius salei* (Keyserling, 1877) wandering spiders dive into the bromeliad water *Aechmea bracteata* (Sw.) Griseb. when it is threatened (Hénaut et al., 2018). The behaviour of submerging in accumulated water in tanker bromeliads is an adaptation for species living in phytotelm (Romero et al., 2007). In addition, the *T. neglectus* diving behaviour may indicate that this scorpion may be a predator of bromeliad aquatic prey. Recently, Piccoli et al. (2024) showed that the spider *Corinna demersa* Rodrigues & Bonaldo, 2014, captures prey in both terrestrial and aquatic bromeliad compartments. The diving behaviour in *T. neglectus* increases the possibilities of studies encompassing Neotropical scorpion natural history, in which the evolutionary mechanisms of this behaviour may be considered for future studies.

## 5 CONCLUSIONS

In summary, our study showed that bromeliad's architecture plays a key role in the likelihood of *T. neglectus* occurrence. It suggests that animals have the ability to choose bromeliads with structures that maximize their survival. In addition, the uniform distribution pattern presented by the species may be explained by competition for resources such as ideal bromeliads, reflecting on high aggressiveness and territoriality presented by scorpions. Finally, *T. neglectus* when threatened shows the capacity to dive in the water accumulated in bromeliads.

## AUTHOR CONTRIBUTIONS

**Maria Carolina de Oliveira Souza:** Data curation (equal); investigation (equal); writing – original draft (equal); writing – review and editing (equal). **Stênio Ítalo Araújo Foerster:** Data curation (equal); formal analysis (equal); funding acquisition (equal); investigation (equal); methodology (equal); validation (equal); visualization (equal); writing – review and editing (equal). **Renato Portela Salomão:** Data curation (equal); formal analysis (equal); validation (equal); visualization (equal); writing – review and editing (equal). **João Pedro Souza‐Alves:** Formal analysis (equal); methodology (equal); validation (equal); writing – review and editing (equal). **Geraldo Jorge Barbosa de Moura:** Conceptualization (equal); funding acquisition (equal); project administration (equal); resources (equal); writing – review and editing (equal). **André Felipe de Araujo Lira:** Conceptualization (equal); data curation (equal); investigation (equal); methodology (equal); validation (equal); writing – review and editing (equal). **Rodrigo Barbosa Ferreira:** Investigation (equal); validation (equal); visualization (equal); writing – review and editing (equal).

## FUNDING INFORMATION

AFAL was supported by Dirección General de Asuntos del Personal Académico (DGAPA) postdoctoral fellowship from the Universidad Nacional Autónoma de México. SÍAF was supported by the Estonian Research Council (PRG741).

## CONFLICT OF INTEREST STATEMENT

The authors declare that they have no conflict of interest.

## Supporting information


Data S1:

## Data Availability

The dataset used in this study is available in Supplementary Material.
